# Optimization of Reservoir Flood Control Operation Based on Multialgorithm Deep Learning

**DOI:** 10.1155/2022/4123421

**Published:** 2022-05-10

**Authors:** Bowen Xue, Yan Xie, Yanhui Liu, Along Li, Daguang Zhao, Haipeng Li

**Affiliations:** ^1^School of Water Conservancy, North China University of Water Resources and Electric Power, Zhengzhou, Henan 450046, China; ^2^Yellow River Institute of Hydraulic Research, Zhengzhou, Henan 450003, China; ^3^College of Water Conservancy and Hydropower Engineering, Hohai University, Nanjing, Jiangsu 210098, China; ^4^Yellow River Engineering Consulting Co., LTD, Zhengzhou, Henan 450003, China; ^5^Jilin Province Water Conservancy and Hydropower Survey and Design Institute, Changchun, Jilin 130021, China; ^6^College of Agricultural Science and Engineering, Hohai University, Nanjing, Jiangsu 210098, China; ^7^Technical Advisory of PRWRC (Guangzhou) Co., Ltd, Guangzhou 510000, China

## Abstract

With the rapid development of China's social economy, it is the most important task for the water conservancy industry to make use of the existing water conservancy engineering measures to carry out the research on river basin flood control dispatching. Large-scale joint operation of river basins usually needs to consider meteorological and hydrological conditions, historical flood data, multireservoir engineering conditions, and multiple flood control targets, which is a complex decision-making problem. Therefore, electing the optimal operation model of reservoir flood control optimization is very important. In this paper, Luanhe River Basin is taken as the research area, and three kinds of constraints, namely, water balance constraint, reservoir flood control capacity constraint, and water release decision constraint, are set to construct the flood control optimization model. Taking the minimum square of the sum of reservoir discharge and interval flood discharge as the objective function, genetic algorithm (GA), particle swarm optimization (PSO), Spider swarm optimization (SSO), and grey wolf optimization (GWO) are introduced into flood control optimal operation to seek the minimum value of objective function, and the results are compared and analyzed. Through the analysis of optimization results, the optimization ability and convergence effect of grey wolf optimization algorithm are better than those of genetic algorithm and particle algorithm, and the results are more stable than those of spider swarm algorithm. It has a good model structure and can make full use of the results of three wolf groups for optimization. Through the analysis of scheduling results, the results of genetic algorithm and particle swarm optimization algorithm are similar, while those of spider swarm optimization algorithm and grey wolf optimization algorithm are similar and slightly better than those of the first two. Moreover, the search range of grey wolf optimization algorithm for solving long sequence problems is wider and the calculation time is shorter. Therefore, the grey wolf optimization algorithm can be applied to solve the flood control operation optimization model of Panjiakou Reservoir Group.

## 1. Introduction

With the rapid development of China's social economy, the impact of human activities on the environment is aggravated, and the economic losses caused by floods account for the first of all kinds of natural disasters in China. How to make use of the existing water conservancy project measures and scientifically carry out the research on flood control dispatching in river basins is the most important task of disaster prevention [1]and mitigation in water conservancy industry at present. When guiding the flood control operation of reservoir groups, it still depends more on the experience of decision-making level. There are still some problems when the existing mathematical models and scientific theories are applied to the actual operation [2, 3]. With the development of modernization, more and more reservoirs with flood control functions have been built in large and medium-sized river basins, and the research on joint optimal operation of reservoir groups has become one of the hot topics. In the past reservoir operation, some scholars [4] showed that the joint operation model of cascade reservoirs is constructed, considering the uncertain inflow from the upstream of reservoirs, and the Copula function is used for modeling, which has achieved remarkable results. On this basis, particle swarm optimization is adopted [5]. Increase the calculation speed and improve the algorithm [6]. Improving the global optimization ability makes it possible to use mathematical analysis method to formulate a scientific dispatching scheme for reservoir dispatching and give better play to the role of water conservancy projects.

The intelligent optimization algorithm is mostly used in the optimization of reservoir flood control optimal operation model. With the deepening of research, the complexity of reservoir flood control optimal operation is gradually excavated, and the mathematical model based on this is nonlinear, multiobjective, and high-dimensional [7]. In order to improve the efficiency and accuracy of model solution [8], traditional optimization methods often fail to meet the complex requirements of new problems [9]. Therefore, more and more optimization algorithms have been developed and utilized by scholars in flood control optimal operation, such as genetic algorithm [10, 11]. The algorithm based on evolutionary thinking uses natural evolutionary rules to achieve iterative optimization. Based on spider swarm algorithm [12], by simulating the social behavior of social spiders in the natural environment, the optimization target is drawn up as the real object on the spider web, and the female and male spiders are drawn up as two optimization methods and search for “food” on the spider web according to their different standards. Wolves algorithm [13] mainly simulates the behavior of wolves searching for prey, surrounding prey, and attacking prey [14]. On the basis of conventional dispatching theory, the combined optimal dispatching of reservoirs uses advanced intelligent optimization algorithm and system science concept to solve the optimal strategy which meets the operation requirements of reservoirs in the basin [15], so as to obtain the benefits that are difficult to realize when a single reservoir is operated. Large-scale joint operation of river basins usually needs to consider meteorological and hydrological conditions, historical flood data, multireservoir engineering conditions, and multiple flood control targets, which is a complex decision-making problem [16]. According to the characteristics of Luanhe River basin and flood control requirements, this paper discusses the optimal dispatching model of reservoir flood control optimization based on multialgorithm deep learning.

**Figure 1:**
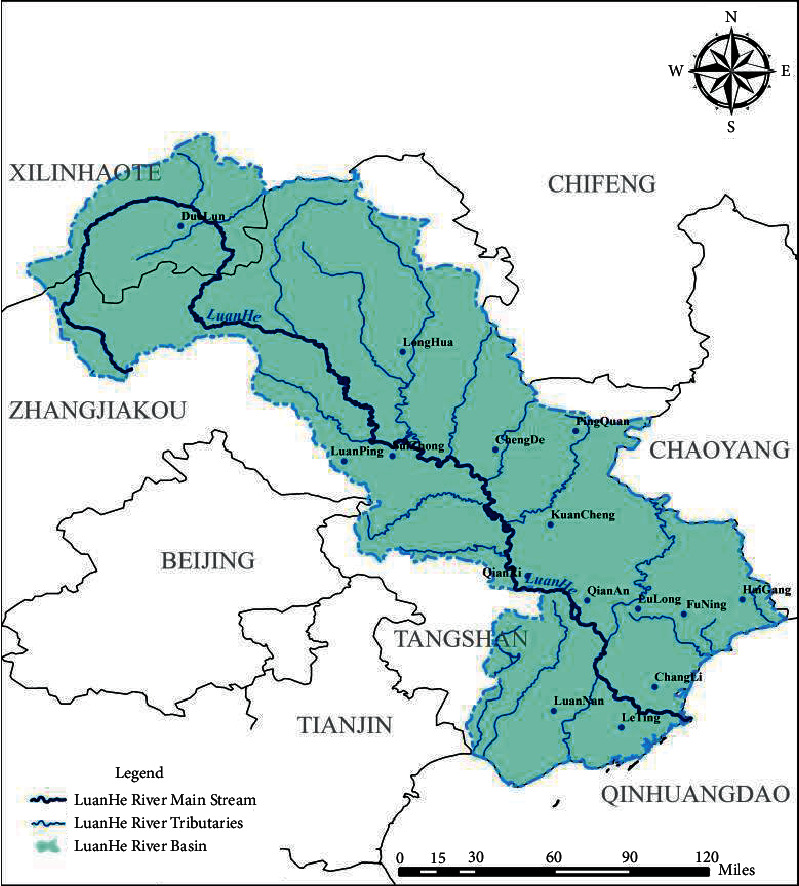
The drainage map of Luanhe River Basin.

The latitude and longitude range of Luanhe River Basin is 115°30′∼119°45′ east longitude, 39°10′∼42°40′ north latitude, the northern end to the southern edge of Inner Mongolia Plateau, the intersection with Bohai Sea in the south, Chaobai and Jiyun Canal in the west, Liaohe River in the east, and the basin area of 44750 km^2^. Panjiakou, Daheiting, and Taolinkou reservoirs are the three most important large-scale water conservancy projects in Luanhe River Basin. Among them, Panjiakou and Daheiting reservoirs are the main flood control projects on the main stream of Luanhe River, and the controlled basin area accounts for more than 75% of the total area of Luanhe River. Taolinkou Reservoir is the main flood control project on Qinglong River, a tributary, and these three reservoirs control 90% of the area of Luanhe River. The birthplace of Luanhe River is mainly grassland, with a gentle and open terrain, with an average elevation of 1350 m. The slope of riverbed is about 0.0005. Below Panjiakou Reservoir, the river is 200∼500 m wide. After flowing through Sangyuan Canyon, the river widens greatly. After going down to Jingshan Railway Bridge in Luanxian County, the river flows into the plain [17–19]. Luanhe River Basin belongs to a relatively independent water system, and its source flows into the lower main stream and then into the ocean. Considering the length of the river in the lower reaches of the plain terrain area, there is no interval for flood storage, so flood prevention mainly depends on precaution and small dam. See Figure 1 for the schematic diagram of Luanhe River Basin.

## 2. Materials and Methods

### 2.1. Model Building

#### 2.1.1. Objective Function

Setting an appropriate objective function according to the current situation of the basin and the characteristic value of the reservoir is one of the keys to determine the results of flood control optimal operation. Aiming at Panjiakou Reservoir Group in Luanhe River Basin, the joint optimal operation model takes Luanxian Station in the lower reaches of Luanhe River as the control point, and the flow of the control point is mainly composed of three parts: the discharge of Panjiakou Reservoir after flood calculation, Taolinkou Reservoir after flood calculation, and flood in Pan-Tao-Luanhe section, and the discharge of Luanxian Station is a combined discharge process of the above three parts.

There are many objectives for optimal operation of reservoir group joint flood control. At present, the maximum peak clipping criterion and the minimum disaster duration criterion are widely used. In this paper, the maximum peak clipping criterion is used as the objective function of reservoir group joint flood control operation, and the peak clipping rate is used as the evaluation criterion of operation results. The objective function is as follows:

(1) $$\begin{array}{c} F=\min\sum_{t=1}^{n}{\left(q_{\text{pan }t+\Delta t_{1}}+q_{\text{tao }t+\Delta t_{2}}+Q_{\text{qu }t+\Delta t_{3}}\right)}^{2}. \end{array}$$

In the formula, *t* is the serial number of time slots, and N is the total number of scheduled time slots; Δ*t*_1_ is the propagation time (h) from Panjiakou Reservoir discharge to the control section; Δ*t*_2_ is the propagation time (h) from the discharge of Taolinkou Reservoir to the control section; Δ*t*_3_ is the propagation time (h) from flood in Pan-Tao-Luanhe section to control section; *q*_pan*t*_ is water discharge after regulation and storage by Panjiakou Reservoir (m^3^/s); *q*_tao*t*_ is water discharge after regulation and storage by Panjiakou Reservoir (m^3^/s); *Q*_qu*t*_ is Pan-Tao-Luanhe section flood discharge (m^3^/s).

#### 2.1.2. Model Constraints

After the Luanhe River flood control system was completed, it experienced several floods in 1962, 1989, 1994, 2012, etc. Considering the problems existing in flood control in Luanhe River Basin, three kinds of constraint targets for reservoir flood control were set. They are water balance constraint, reservoir flood control capacity constraint, and water release decision constraint.

Water balance constraint:

(2) $$\begin{array}{c} v_{t}=v_{t-1}+\left(q_{i1}-q_{i2}\right)\Delta t. \end{array}$$

Flood control capacity constraints:

(3) $$\begin{array}{c} \sum_{i=1}^{n^{\prime }}\left(q_{i_{1}}-q_{i_{2}}\right)\Delta t\leq v_{3}. \end{array}$$

Decision-making constraints:

(4) $$\begin{array}{c} q_{i_{1}}\leq q_{i_{2}},q_{i_{2}}\leq q\left(z_{t},b_{t}\right). \end{array}$$

Type: *v*_*t*_, *v*_*t*−1_ is reservoir capacity at the beginning and end of the period (m^3^); Δ*t* is length of time period (h); *z*_*t*_ is the water level of the reservoir at time (m); *b*_*t*_ is flood discharge capacity of the reservoir (m^3^/s).

### 2.2. Comparison and Selection of Model Solution Methods

There are two key problems in the study of optimal operation of reservoir group joint flood control: 1. How to build a mathematical model that accords with the reality of the research object; 2. Find an effective algorithm to solve the model.

Genetic algorithm (GA) [20], particle swarm optimization (PSO) [21], spider swarm optimization (SSO) [21], and grey wolf optimization algorithm (GWO) [22], which are among the intelligent optimization algorithms, are applied to solve the Panjiakou Reservoir Group's joint flood control optimal operation model, and the efficiency and optimal operation results of each algorithm are compared and analyzed.

#### 2.2.1. Genetic Algorithm

Genetic algorithm takes the evolution of organisms as its basic idea, and each chromosome is the outflow sequence. It uses real numbers to encode and simulate the crossover, mutation, and selection of chromosomes, but it has the characteristics of premature convergence and difficulty in finding the optimal solution. The core of genetic algorithm is crossover, mutation, and selection. The crossover operator selects the dominant individual based on the fitness of the individual to combine the genes to form a new individual, which can better retain the dominant genes. The mutation operator can make the value of individual gene change randomly, so that the algorithm can generate a new optimal individual and get the global optimal solution. Selecting the fitness is based on the objective function. Choose by roulette, etc.

*(1) Crossover*. The crossover operation used in this paper is the single-point crossover method; that is, a crossover point is randomly selected, and the parts before and after the crossover point of two individuals are combined to form a new individual. *G*_*1*_ and *G*_*2*_ are two discharge sequences, respectively, which produce a crossing point *P* in *G*_*1*_ and *G*_*2*_. The sequence before the crossing point *P* is unchanged, and the sequence after the crossing point is interchanged.

  Before: **G**_**1**_ = {*x*_1_, *x*_2_,., *p*, *x*_*n*−*m*_.., *x*_*n*_}, **G**_**2**_ = {*y*_1_, *y*_2_,., *p*, *y*_*n*−*m*_,.,*y*_*n*_}
  After crossing: **G**_**11**_ = {*x*_1_,*x*_2_,.,*p*,*y*_*n*−*m*_..,*y*_*n*_},**G**_**21**_ = {*y*_1_, *y*_2_,.,*p*,*x*_*n*−*m*_,..,*x*_*n*_}

*(2) Variation*. Variation is based on the variation operator changing a certain value in the discharge sequence to obtain a new discharge sequence. *G* is the discharge sequence, *G* = {*x*_*1*_*, x*_*2*_*, .., p, x*_*n-m*_*.., x*_*n*_}, which changes to *q* at the position of factor *p*, forming *G'* = {*x*_*1*_*, x*_*2*_*, .., q, x*_*n-m*_*.., x*_*n*_}. If the fitness function of the formed sequence is better, the variation will be retained and passed on to the next generation.

*(3) Selection*. Selection is to pass on a better individual to the next generation. The roulette method is used in this paper to select the individual with the smallest fitness function. The fitness of an individual is converted into probability, which is the probability that the individual will be passed on to the next generation. The individual is selected based on the calculated probability and random number.

(5) $$\begin{array}{c} p_{i}=\frac{1/F\left(i\right)}{\sum_{i=1}^{n}1/F\left(i\right)}\left(i=1,2,\ldots ,n\right),\\ P_{i}=P_{i-1}+p_{i}, \end{array}$$

where *F(i)* is fitness of the discharge sequence, the number of samples of *n*-discharge sequence; *P*_*i*_ is the probability that the fitness of the *i-th* sequence accounts for all sequences; *P*_*i*_ is the cumulative sum of the probabilities of the *i-*1*st* sequence to the *i-th* sequence.

**Figure 2:**
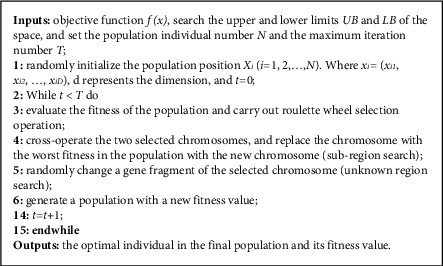
The pseudocode of genetic algorithm.

See the detailed development process of genetic algorithm and the pseudocode of genetic algorithm as shown in Figure 2.

#### 2.2.2. Particle Swarm Optimization

Particle swarm optimization (PSO) is based on the simulation of the behavior of birds flying and preying on groups, and it searches for the best individual based on the evolutionary selection of the best individual of groups and individuals. In the search range, each particle has two characteristics: speed and position, and the particle constantly updates its position and speed by learning the optimal position of the particle itself and the optimal position of the group, so as to search for the best individual. The core of particle swarm optimization algorithm is the update and iteration of speed and position.

Update and iteration of speed:

(6) $$\begin{array}{c} V_{i}\left(j+1\right)=\zeta V_{i}\left(j\right)+\delta_{1}\rho_{1}\left(pB_{i}\left(j\right)-X_{i}\left(j\right)\right)+\delta_{2}\rho_{2}\left(gB\left(j\right)-X_{i}\left(j\right)\right). \end{array}$$

Update and iteration of location:

(7) $$\begin{array}{c} X_{i}\left(j+1\right)=X_{i}\left(j\right)+V_{i}\left(j+1\right), \end{array}$$

where *V*_*i*_ is the velocity vector of the *i*th particle, with *m* elements (*v*_*i*_^1^, *v*_*i*_^2^,…, *v*_*i*_^*m*^); *X*_*i*_ is the position vector of the *i*th particle, with *m* elements (*x*_*i*_^1^, *x*_*i*_^2^,…, *x*_*i*_^*m*^);*ζ* is inertia weight; *j* is number of iterations; *δ*_1_, *δ*_2_ are learning factors, used to characterize the importance of *PB*_*i*_, *gB*; *ρ*_1_, *ρ*_2_ are random numbers on the interval; *pB*_*i*_, *gB* are the best position to separate particle *i* from group particles.

**Figure 3:**
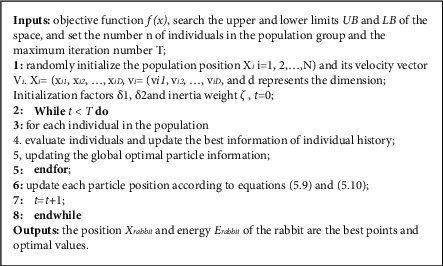
The pseudocode of particle swarm optimization algorithm.

See detailed development process of particle swarm optimization and pseudocode of particle swarm optimization as shown in Figure 3.

#### 2.2.3. Spider Swarm Algorithm

Spider swarm optimization algorithm is to simulate the information exchange, mating, and selection of male and female spiders. Spiders transmit information through vibration on the spider web. Spiders are divided into male and female. The male and female spiders transmit information based on the vibration model. The dominant male spiders mate with the female spiders and protect the whole population for foraging. Information exchange and mating behavior of male and female spiders are the core of spider algorithm.

*(1) Algorithm initialization*. Initialization of spider group, including the number of male and female spiders, position, and weight initialization is as follows:

(8) $$\begin{array}{c} N_{f}=\text{floor}\left[\left(0.9-\text{rand}\cdot 0.25\right)\cdot N\right],N_{m}=N-N_{f},\\ s\left(i\right)=LB+\text{rand}\left(1,D\right)\cdot UB,\\ \omega \left(i\right)=\frac{J\left(X\left(i\right)\right)-\text{worst}\left(X\right)}{\text{best}\left(X\right)-\text{worst}\left(X\right)}. \end{array}$$

In the above formula, n_*f*_ and n_*m*_ are the numbers of female and male spiders, *n* is the population review, rand is the random number generated by uniform distribution [0,1], *LB* and *UB* are the upper and lower limits of the search space, and floor is the rounding function, *ω* is the individual weight, *J (s (I))* is the fitness value, best (*s*) is the optimal fitness value, and worst (*s*) is the worst fitness value.

*(2) Information exchange.*

(a) The mathematical model of the vibration perception ability of individual *J* to individual I is as follows:
  (9) $$\begin{array}{c} v_{i,j}=\omega_{j}\times e^{-l_{i,j}^{2}}. \end{array}$$
Type: *v*_*i*,*j*_ is information on the female nearest to spider I(*v*_*i*,*f*_), the information of the best spider nearest to spider I(*v*_*i*,*b*_), and information of individuals closest to spider *I* and with larger weight (*v*_*i*,*c*_);*ω*_*j*_ is individual weight; *d*_*ij*_ is the distance between I and *j* of spiders.
(b) Vibration patterns of female spiders:
  (10) $$\begin{array}{c} f_{i}\left(j+1\right)=\left\{\left\{\begin{array}{cc} f_{i}\left(j\right)+\delta_{1}\times v_{i,c}\left(P_{c}-f_{i}\left(j\right)\right)+\delta_{2}\times v_{i,b}\left(P_{b}-f_{i}\left(j\right)\right)+\delta_{3}\times \left(\text{rand}-0.5\right), & \delta_{4}<PF\\ f_{i}\left(j\right)-\delta_{1}\times v_{i,c}\left(P_{c}-f_{i}\left(j\right)\right)-\delta_{2}\times v_{i,b}\left(P_{b}-f_{i}\left(j\right)\right)+\delta_{3}\times \left(\text{rand}-0.5\right), & \delta_{4}\geq PF \end{array}\right.\right.,\\ PF=\exp\left(-pf_{i}\right). \end{array}$$
Type: *δ*_1_, *δ*_2_, *δ*_3_, *δ*_4_ are random numbers in the interval of and Rand[0,1]; *j* is the number of iteration cycles; *P*_*c*_is individuals with the smallest distance and larger weight from spiders *I*; *P*_*b*_ is individuals with the smallest distance and larger weight from spiders *I*; *Pf* is probability factor used to control the attraction and repulsion behavior of spiders.
(c) Vibration patterns of male spiders: male spiders can automatically be identified and gathered, which can be divided into dominant male spiders and nondominant male spiders. Dominant spiders can attract female spiders, and nondominant spiders move towards dominant spiders. The movement of nondominant spiders to dominant spiders can be expressed as follows:
  (11) $$\begin{array}{c} m_{i}\left(j+1\right)=\left\{\begin{array}{cc} m_{i}\left(j\right)+\delta_{1}\times v_{i,f}\left(P_{f}-m_{i}\left(j\right)\right)+\delta_{3}\times \left(\text{rand}-0.5\right), & \text{if }\left(\omega_{N_{f+i}}>\omega_{N_{f+m}}\right),\\ m_{i}\left(j\right)+\delta_{1}\times \left(\frac{\sum_{h=1}^{N_{m}}m_{h}^{j}\times \omega_{N_{f+h}}}{\sum_{h=1}^{N_{m}}\omega_{N_{f+h}}}\right), & \text{if }\left(\omega_{N_{f+i}}\leq \omega_{N_{f+m}}\right). \end{array}\right. \end{array}$$
In the above formula, *P*_*f*_ is the female nearest to the dominant male; *ω*_*N*_*f*+*m*__ is the weight of spiders in the middle; ∑_*h*=1_^*N*_*m*_^*m*_*h*_^*j*^ × *ω*_*N*_*f*+*h*__/∑_*h*=1_^*N*_*m*_^*ω*_*N*_*f*+*h*__ is middle position of male spider; *ω*_*N*_*f*+*i*__ > *ω*_*N*_*f*+*m*__ is the distinguishing conditions of dominant male spiders; *ω*_*N*_*f*+*i*__ ≤ *ω*_*N*_*f*+*m*__ is the discriminant conditions of nondominant male spiders.

*(3) Mating Behavior*. Male dominant spiders will mate with female spiders within the mating radius. If there are more female spiders within the mating radius, roulette will be used to produce new spiders.

Mating radius can be calculated by the following formula:

(12) $$\begin{array}{c} r=\frac{\sum_{k=1}^{n}\left(p_{k}^{h}-p_{k}^{l}\right)}{2n}. \end{array}$$

Type: *p*_*k*_^*h*^, *p*_*k*_^*l*^ are the upper and lower limits of the *k*-th dimensional variable of the discharge sequence.

**Figure 4:**
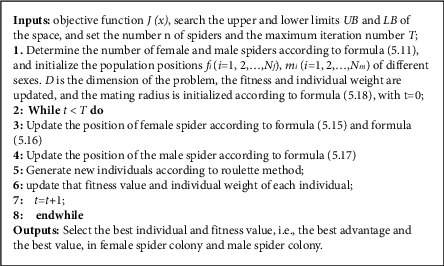
The pseudocode of particle social-spider optimization algorithm.

See the detailed development process of spider swarm algorithm and the pseudocode of spider swarm algorithm as shown in Figure 4.

#### 2.2.4. Grey Wolf Optimization Algorithm

The algorithm is based on the division of labor and information interaction in wolves' predation to realize the search of the optimal solution. Wolves are divided into *α*-wolf, *β*-wolf*, δ*-wolf, and other wolves. *α*-Wolf*, β*-wolf, and *δ*-wolf are the leader wolves of the wolves, representing the optimal solution in evolution, and other wolves do not know the information of the optimal solution. Other wolves constantly adjust the search range and step size based on the position of the leader wolf, and the three best wolves formed after adjustment will become the leader wolves, so as to search for the best solution through continuous loop iteration. The algorithm simulates the encirclement, hunting, attack, and search of wolves.

*(1) Surround*. The positions of other wolves can be represented by the following formula:

(13) $$\begin{array}{c} D=\left|C\cdot X_{q}\left(k\right)-X\left(k\right)\right|,\\ X\left(k+1\right)=X_{q}\left(k\right)-A\cdot D,\\ A=2\zeta \cdot \tau_{1}-\zeta ,C=2\tau_{2}. \end{array}$$

Type: *X*_*q*_ is the position of prey, which can be replaced by the mean value of *α*, *ß*, and *δ* wolves; *X*(*k*) is the K-generation individual wolf pack; *τ*_1_, *τ*_2_ are random numbers in the interval [0,1]; *ζ* is linearly decreasing from 2 to 0 based on the number of iterations.

*(2) Hunting*. In this step, it is assumed that *α*-wolf, *β*-wolf, and *δ*-wolf know the position of prey (optimal solution), and the algorithm uses these three positions to calculate the optimal solution. *D*_*ρ*_=|*C*_*ρ*_ · *X*_*ρ*_ − *X*(*k*)|, *ρ*=*α*, *β*, *δ*. At the same time, other wolves are approaching the optimal solution.

The positions of *α*-wolf*, β*-wolf, and *δ*-wolf can be represented by the following formula:

(14) $$\begin{array}{c} X_{\rho }^{\prime }=X_{\rho }-A_{\rho }\cdot D_{\rho },\\ X\left(k+1\right)=\frac{X_{\alpha }^{\prime }+X_{\beta }^{\prime }+X_{\delta }^{\prime }}{3}. \end{array}$$

Type: *D*_*ρ*_ is the distance between *α*, *β*, and *δ* wolves and other individuals; *X*_*ρ*_ is the position of *α, β*, and *δ* wolves; *X*(*k*) is the location of other individual wolves; *X*_*ρ*_′ is the position of other individual wolves moving towards *α, β*, and *δ* wolves.

*(3) Attack*. Through the continuous iteration of the algorithm, *α, β*, and *δ* wolves approach the optimal solution continuously, achieving the purpose of optimization. In the simulation of this process, *ζ* is linear decrease, from 2 to 0, and the range of a value is [−*ζ*, *ζ*]. When |A|<1, the next position of the wolf pack will be closer to the position of the prey, and the algorithm will search locally. When |A|>1, the distance between the next position of the wolf pack and the position of the prey becomes larger, and then the algorithm performs global search.

*(4) Search*. Individual wolves search for the best solution (prey) based on the position of *α, β*, and *δ* wolves. The algorithm is based on the relationship between |A| value and 1 as the standard to divide global search and local search. In order to avoid the search falling into the local optimal solution, the grey wolf algorithm introduces the parameter *C.* The parameter *C* is a random value in the interval [0,2] and decreases nonlinearly. *C* > 1 indicates that the random weight of the position of the wolf pack is significant, and *C* < 1 indicates that the random weight of the position of the wolf pack is small. Parameter *c* can make the algorithm search globally and can make the algorithm jump out of the local optimal solution.

**Figure 5:**
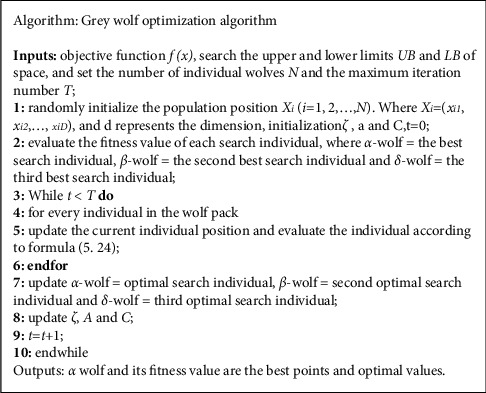
The pseudocode of wolf optimization algorithm.

See the detailed development process of the grey wolf algorithm and the pseudocode of the grey wolf algorithm as shown in Figure 5.

### 2.3. Selection of Optimization Methods

#### 2.3.1. Solution Method

Based on the analysis of flood composition in Luanhe River Basin, taking the minimum square of the sum of reservoir discharge and interval flood discharge as the objective function, four optimization algorithms, namely, genetic algorithm (GA), particle swarm optimization (PSO), spider swarm optimization (SSO), and grey wolf optimization algorithm (GWO), are introduced into the optimal flood control operation to seek the minimum value of the objective function, and the results are compared and analyzed.

#### 2.3.2. Introduction of Parameters

The computer is used for simulation experiment. The selected computer is Win10 and 64-bit operating system, the processor is 2.9 GHz, and the memory is 16 GB. MATLAB2018b is used for programming calculation. The number of samples is 50 and the maximum number of iterations is 500. The optimized parameters of the algorithm are obtained by experiment and debugging: the crossover probability of genetic algorithm is 0.8, and the mutation probability is 0.2; inertia weight of particle swarm optimization is *ζ*. It is a linearly decreasing value in the range of 0.9∼0.4, *δ*_1_=*δ*_2_=2. The probability factor *pfi* of spider algorithm is 0∼3 increasing value. The parameters of the grey wolf optimization algorithm have been explained above. This paper analyzes the optimization algorithm of flood dispatching in Panjiakou Reservoir and finds out the optimal method for optimal flood dispatching in Luanhe River Basin.

## 3. Results and Discussion

### 3.1. Optimization Results

Genetic algorithm, particle swarm optimization, spider swarm optimization, and grey wolf optimization algorithm are applied to the optimal flood control operation of Panjiakou Reservoir, and the abovementioned models are applied to the optimal flood control operation of the basin once every 3 years, once every 5 years, once every 10 years, once every 20 years, and once every 50 years. The change of the result of each iteration of the optimization algorithm with the number of iterations is taken as the basic basis for analyzing the performance of the optimization algorithm.

**Figure 6:**
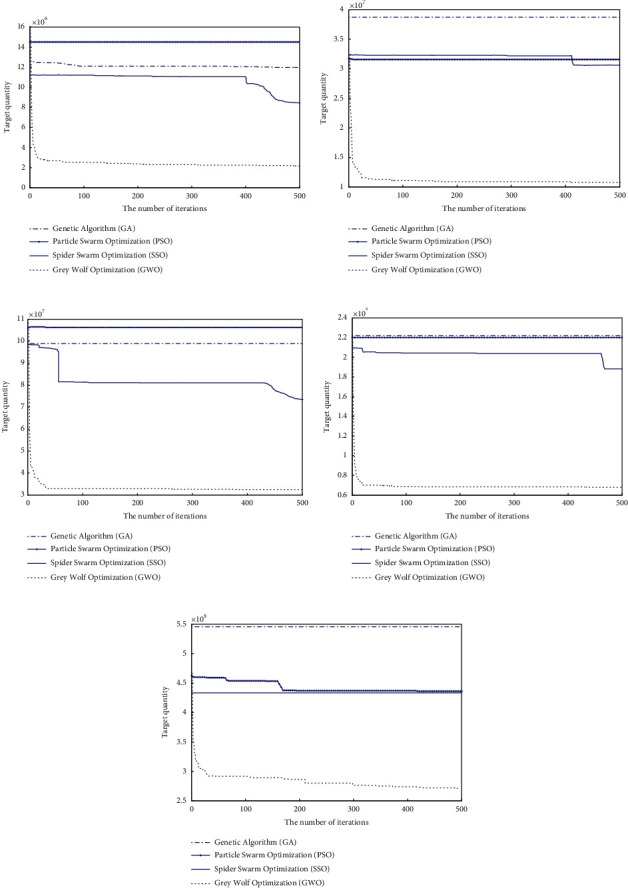
Iterative process of Panjiakou Reservoir operation with 3-year return period (a), 5-year return period (b), 10-year return period (c), 20-year return period (d), and 50-year return period (e).

From the analysis in Figure 6, it can be seen that, for the flood that occurs once every three years or once every five years, the grey wolf optimization algorithm is the best iteration of the four methods, and the grey wolf optimization algorithm, genetic algorithm, and particle swarm algorithm converge earlier, and the best convergence value of the grey wolf optimization algorithm is better than that of genetic algorithm and particle swarm algorithm. Spider swarm optimization algorithm converges late, but the optimal value of convergence is better than genetic algorithm and particle swarm optimization algorithm. For the once-in-ten-year flood, the best iteration of the four methods is the grey wolf optimization algorithm, which converges earlier than the spider swarm algorithm and the particle swarm algorithm, and the best convergence value of the grey wolf optimization algorithm is better than that of the spider swarm algorithm and the particle swarm algorithm. Spider swarm algorithm converges later than spider swarm algorithm, and the objective function value of genetic algorithm changes little. According to the change trend in the figure, with the increase of iteration times, the spider swarm algorithm may continue to converge.

For the flood that occurs once every 20 years, the analysis shows that the change trend of genetic algorithm and particle swarm optimization is similar, particle swarm optimization is slightly better than genetic algorithm, and the convergence trend of genetic algorithm and particle swarm optimization is not obvious. The convergence performance of grey wolf optimization algorithm and spider swarm algorithm is better, and the convergence performance of grey wolf optimization algorithm is better than that of spider swarm algorithm. By analyzing the changing trend of the objective function, it can be seen that the spider swarm algorithm may still converge with the increase of iteration times.

For the once-in-50-year flood, the analysis shows that the convergence performance of genetic algorithm and particle swarm optimization is not obvious and basically maintains the initial value. The convergence performance of spider swarm algorithm and grey wolf optimization algorithm is better than that of spider swarm algorithm. Spider swarm optimization is basically stable after convergence reaches a certain objective function value. The grey wolf optimization algorithm converges faster and better. Based on the changing trend, it is known that the grey wolf optimization algorithm still has the possibility of continuous iterative convergence.

### 3.2. Scheduling Results

The results of reservoir operation are evaluated by the sum of squares of outflow and peak clipping rate, and the results of flood control optimal operation are analyzed and calculated. The results are shown in Table 1. As can be seen from Table 1, for the analysis of the sum of squares of the outflow, the spider swarm algorithm and the grey wolf optimization algorithm of the inflow flood once every 3 years, 5 years, 10 years, and 20 years are superior to the genetic algorithm and the particle swarm algorithm. The grey wolf optimization algorithm is the best. The 50-year return spider swarm algorithm and particle swarm algorithm have similar results, the worst is genetic algorithm, and the best is grey wolf optimization algorithm. According to the analysis of peak clipping rate of the inflow flood with the frequency of 5 years, 10 years, 20 years, and 50 years, the order of peak clipping performance from high to low is grey wolf optimization algorithm > spider swarm algorithm > particle swarm algorithm > genetic algorithm. For the flood that occurs once every three years, the order of peak clipping performance from high to low is grey wolf optimization algorithm > particle swarm algorithm > spider swarm algorithm > genetic algorithm.

## 4. Conclusions

Genetic algorithm (GA), particle swarm optimization (PSO), spider swarm optimization (PSO), and grey wolf optimization (GWO) are applied to the optimal operation of reservoir flood control.

1. Optimization ability: the analysis shows that genetic algorithm and particle swarm optimization algorithm have obvious premature convergence characteristics and poor iterative optimization performance. The spider swarm algorithm has strong optimization ability, and its optimization ability has strong correlation with iteration times and optimization time. Grey wolf optimization algorithm has strong optimization ability, good convergence effect, and stable results. Analysis shows that grey wolf optimization algorithm is better than spider swarm algorithm for long-term flood control operation. There are two types of spiders, male and female, and grey wolf optimization algorithm *α*, *β*, and *δ* wolves and other types of wolves are the main particles for optimization. It shows that dividing the artificial intelligence population into different types of elite particles can expand the search range and search ability from different angles, so as to effectively improve the optimization ability of the algorithm. The simulation formula of male and female spiders of spider swarm algorithm is complex, while the simulation of grey wolf optimization algorithm is simple and easy to understand and operate, which shows that grey wolf optimization algorithm has a good model structure. It can make full use of the results of the three wolf groups to optimize.
2. Scheduling results: from the analysis of the square sum of the outflow flow and the peak clipping rate, the results of genetic algorithm and particle swarm optimization algorithm are similar, and the results of spider swarm algorithm and grey wolf optimization algorithm are similar and slightly better than the first two. Combined with the optimization ability, it can be expressed as follows: the algorithm with better optimization performance has strong peak clipping ability and has good peak clipping ability for each period in the iterative calculation process. The peak clipping rate of grey wolf optimization algorithm is more stable than spider swarm algorithm.

Based on the above analysis, it can be seen that grey wolf optimization algorithm has better optimization performance, and the Panjiakou Reservoir group data series is longer. Grey wolf optimization algorithm has a wider search range and shorter calculation time in solving the long sequence problem. Therefore, grey wolf optimization algorithm can be applied to solve the flood control operation optimization model of Panjiakou Reservoir Group.

## Figures and Tables

**Table 1:** Statistical table of inflow flood regulation results.

Eigenvalue	Way	Once in three years	Once in five years	Once in 10 years	Once in 20 years	Once in 50 years
*Sum of squares of flow*	Genetic algorithm	5.47 × 10^6^	3.88 × 10^7^	5.91 × 10^7^	8.22 × 10^7^	3.90 × 10^8^
	Particle swarm optimization algorithm	4.25 × 10^6^	3.15 × 10^7^	4.06 × 10^7^	8.20 × 10^7^	3.34 × 10^8^
	Spider swarm algorithm	4.42 × 10^6^	3.06 × 10^7^	4.36 × 10^7^	7.88 × 10^7^	3.37 × 10^8^
	Grey wolf optimization algorithm	3.96 × 10^6^	1.08 × 10^7^	3.26 × 10^7^	6.81 × 10^7^	2.75 × 10^8^

*Peak clipping rate (%)*	Genetic algorithm	59.65	65.62	59.73	63.52	64.09
	Particle swarm optimization algorithm	67.82	61.59	61.32	64.55	65.92
	Spider swarm algorithm	61.98	69.48	64.69	65.69	66.23
	Grey wolf optimization algorithm	70	70	70	70	69.90

## Data Availability

The figures and tables used to support the findings of this study are included in the article.
